# Alteration of CaBP Expression Pattern in the Nucleus Magnocellularis following Unilateral Cochlear Ablation in Adult Zebra Finches

**DOI:** 10.1371/journal.pone.0079297

**Published:** 2013-11-14

**Authors:** Jie Li, Xin Zhou, Li Huang, Xin Fu, Jin Liu, Xinwen Zhang, Yingyu Sun, Mingxue Zuo

**Affiliations:** 1 Beijing Key Laboratory of Gene Resource and Molecular Development, Laboratory of Neuroscience and Brain Development, College of Life Sciences, Beijing Normal University, Beijing, China; 2 Department of Biology, Hainan Normal University, Haikou, China; Rutgers University, United States of America

## Abstract

Songbirds have the rare ability of auditory-vocal learning and maintenance. Up to now, the organization and function of the nucleus magnocellularis (NM), the first relay of the avian ascending auditory pathway is largely based on studies in non-vocal learning species, such as chickens and owls. To investigate whether NM exhibits different histochemical properties associated with auditory processing in songbirds, we examined the expression patterns of three calcium-binding proteins (CaBPs), including calretinin (CR), parvalbumin (PV) and calbindin-D28k (CB), and their relations to auditory inputs in NM in adult zebra finches. We found enriched and co-localized immunostaining of CR, PV and CB in the majority of NM neurons, without neuronal population preference. Furthermore, they were sensitive to adult deafferentation with differential plasticity patterns. After unilateral cochlear removal, CR staining in the ipsilateral NM decreased appreciably at 3 days after surgery, and continued to decline thereafter. PV staining showed down-regulation first at 3 days, but subsequently recovered slightly. CB staining did not significantly decrease until 7 days after surgery. Our findings suggest that the three CaBPs might play distinct roles in association with auditory processing in zebra finches. These results are in contrast to the findings in the NM of chickens where CR is the predominant CaBP and deafferentation had no apparent effect on its expression. Further extended studies in other avian species are required to establish whether the difference in CaBP patterns in NM is functionally related to the different auditory-vocal behaviors.

## Introduction

Songbirds have the rare ability to produce learned vocal behavior, which is found in only a few animal species, including humans [1], [2]. Auditory feedback is essential for vocal learning and maintenance. For example, auditory disturbance or deprivation in songbirds leads to abnormal song learning or plastic changes of the learned songs [3], [4], [5], [6], [7]. Several recent studies in zebra finches and Bengalese finches further showed that songbirds could discriminate subtle changes in the acoustic stimuli (e.g. the frequency) to elaborately modulate their learned songs [8], [9], [10]. A reasonable hypothesis is that songbirds may have special properties in the auditory pathway to adapt to their rare vocal behaviors. A few recent reports indeed have shown that the projection topography details among brainstem auditory nuclei in zebra finches are different from those in barn owls and chickens [11], [12], [13].

The brainstem nucleus magnocellularis (NM) is the first relay of avian ascending auditory pathways and projects bilaterally to the nucleus laminaris (NL). The NM is thought to be equivalent to the mammalian anteroventral cochlear nucleus (AVCN), and considered to be specialized for coding temporal auditory information [14], [15], [16], [17], [18], [19]. Up to now, major histochemical and physiological properties of NM were based on studies in non-vocal learning species, and very few data are available for the NM in songbirds [20]. We chose to investigate the neurochemical properties of songbird NM using antibodies against calretinin (CR), parvalbumin (PV) and calbindin-D28k (CB). These three calcium-binding proteins (CaBPs), which belong to EF-hand family of CaBPs [21], [22], [23], [24], are found to be expressed in functionally distinct neuronal populations in the vertebrate auditory system [22], [23], [24], [25]. In addition, studies in several mammals have shown that the expression of CR, PV or CB in ascending auditory pathways is dependent on auditory inputs, and they respond differently to deafferentation [26], [27], [28], [29]. For example, in guinea pigs, deafferented AVCN neurons showed rapid reduction of CR immunoreactivity, whereas PV staining showed an initial increase followed by sudden decrease in staining. An increase of CB immunoreactivity was observed within deafferented neurons in the medial nucleus of the trapezoid body (MNTB), a higher order region of the auditory brainstem in mammals [26]. Such evidence indicates that the three CaBPs may play certain specific roles associated with auditory processing rather than simply buffering intracellular calcium transients, and serve as useful markers for functional properties of auditory neurons [24], [25].

In the present study in adult zebra finches, we found all three CaBPs co-expressed in the majority of NM neurons, without neuronal population preference. Furthermore, the expression of the three CaBPs was sensitive to deafferentation with differential temporal plastic patterns, although they were largely co-localized. It is interesting to note that these results are different from the reports in the NM of chickens where CR is the predominant CaBP and deafferentation had no apparent effect on its expression [30], [31]. More research in other species is needed to clarify whether the difference in CaBP patterns in NM is functionally related to the different auditory-vocal behaviors.

## Materials and Methods

### Ethics Statement

This study was carried out in accordance with the guidelines of Beijing Laboratory Animal Welfare & Ethics Review. The protocol was reviewed and approved by the Animal Management Committee of College of Life Sciences, Beijing Normal University.

### Animals and Surgery

All birds were purchased from a local supplier and kept in our laboratory with free access to food and water. A total of 36 adult male birds at about 4 months of age were used in this study, in which 15 birds underwent surgery for unilateral cochlear removal. For each lesion case, only the left cochlea was removed. After the bird was anesthetized with sodium pentobarbital (60 mg/kg), a small incision was made in a small area of skin and the tympanic membrane overlaying the middle ear cavity was removed. A fine wire hook was then inserted through the oval window, and the cochlea was pulled out. The extracted cochlea was verified to be complete under a dissecting microscope. The birds were analyzed at 3, 7 or 14 days after surgery (5 birds in each group). Three additional birds underwent sham operations to control any potential effects of the surgery per se. Sham operations consisted of the same anesthetic protocol and skin incision as the unilateral cochlear removal birds above, but without extraction of the tympanic membrane and cochlea [32]. These 3 birds were analyzed at 14 days after operation. No differences in CaBP immunostaining and histological measurements, including the nucleus volume and cross-sectional area of neurons in NM were observed between sham operated and intact birds (n = 5), indicating that the surgery itself has no measurable effect on these parameters. Therefore, the sham operated birds together with the intact birds are henceforth referred to as control group.

### Western Blot Analysis

The specificity of the antibodies for CR, PV and CB was examined by Western blot analysis. Fresh whole brain tissue was collected from an adult zebra finch and homogenized and lysed in sample buffer containing 1% SDS. Samples were boiled and then loaded onto 12% SDS-PAGE gels. The samples were separated and electro-transferred onto PVDF membranes (Millipore). Membranes, blocked with TBST (10 mM Tris–HCl, pH 7.5, 150 mM NaCl, 0.1% Tween-20) containing 5% nonfat dry milk for 1 h, were incubated overnight at 4°C in a solution containing the primary antibodies to CR (1∶3,000, 7699/3H, Swant), PV (1∶5,000, MAB1572, Millipore) or CB (1∶3,000, 38a, Swant). Membranes were rinsed, incubated with an HRP-conjugated goat anti-rabbit (1∶5,000 for CR, 1∶10,000 for CB, Jackson) or goat anti-mouse secondary antibody (1∶5,000, Jackson) for 1.5 h at room temperature, and detected with enhanced chemiluminesence reagents (ECL, Millipore).

### Nissl Staining and Immunohistochemistry

After being anesthetized with a lethal dose of Urethane (1.25 g/kg), birds were perfused transcardially with 0.9% saline, followed by fresh ice-cold 4% paraformaldehyde (PFA) in phosphate buffer (PB, 0.1 M, pH 7.4). The brains were removed and post-fixed for 5–6 h, then placed in 30% sucrose in 0.1 M PB (pH 7.4) at 4°C until sunk. The brains were subsequently sectioned on a freezing microtome cryostat in 10 µm slices in either the coronal or sagittal plane and mounted on gelatin-coated slides as six evenly spaced series. One series was used to determine morphological changes in NM by cresyl violet staining (i.e. Nissl staining), the other five series were used for evaluations of CaBP expression in NM by immunofluorescence staining, including one which served as control.

NeuN (Neuronal Nuclei) was used as a specific neuronal marker [33]. By double immunofluroscence labeling, expression patterns of the three CaBPs in NM were studied. Sections were rinsed in phosphate-buffered saline (PBS, 0.01 M, pH 7.4) and preincubated for 30 min in a blocking solution containing 3% normal goat serum (NGS) in PBS with 0.5% Triton X-100 (TX). They were subsequently incubated with mouse monoclonal antibody against PV (1∶500, MAB1572, Millipore) or NeuN (1∶400, MAB377, Millipore) overnight at 4°C. After 3 rinses in PBS, the sections were incubated with Alexa fluor 594 goat anti-mouse secondary antibodies (1∶200, A11005, Invitrogen) for 2 h. Following extensive rinses in PBS, sections were then transferred into the rabbit primary antiserum against CR (1∶1,000, 7699/3H, Swant) or CB (1∶500, 38a, Swant) overnight at 4°C. On the following day, the sections were incubated at room temperature for 3 h. They were subsequently washed 3 times in PBS and then incubated with Alexa fluor 488 goat anti-rabbit secondary antibodies (1∶200, A11008, Invitrogen) for 2 h. After rinsing in PBS, the sections were covered with antifade solution. Control experiments were carried out using the same procedure as described above, except for the absence of primary antibodies. No immunostaining signal was detected in the control experiments.

### Neuronal Tracing

To further confirm the expression ratios of the three CaBPs in NM, immunostaining of CR, PV or CB combined with retrograde tracing were also performed. Birds were anesthetized with sodium pentobarbital (60 mg/kg) and held in a stereotaxic apparatus (n = 6). About 0.1 µl of Alexa Fluor 488 dextran (10,000 MW, D22910, 5% in PBS, Invitrogen) was used as a retrograde tracer and injected into the NL, the only projection target of the NM [11], through a glass pipette attached to a Nanoject II microinjector (Drummond Scientific). After 7 days, the birds were sacrificed and fixed with PFA, and the brains were cut in three series in sagittal plane in 10 µm sections with a freezing microtome. Then the sections were processed for rabbit anti-CR, mouse anti-PV, rabbit anti-CB immunofluorescence labeling, respectively, according to the staining procedures as described above. The sections were examined with a laser scanning confocal microscope (LSM700, Zeiss), using a high resolution immersion objective (40× oil, NA 1.3, Z-step 500 nm, 1024×1024 pixels).

### Histological Measurements in NM

Every mounted cresyl violet section in a one-in-six series throughout the rostral to caudal extent of NM was examined for histological analysis. Images of NM on both sides of the brain were captured with a digital camera (cool snap, Diagnostic Instruments) attached to the BH microscope (Olympus). The NM on the contralateral side provided a within-animal control.

#### Nucleus volume

The outlines of NM were traced at a lower magnification (20× object) and the surface area enclosed by the outlines was measured with ImageJ v. 1.44 software (NIH Image program). Total volume of the nucleus on each side in each animal was estimated by multiplying the average surface area by the section thickness and the total number of sections [34].

#### Cross-sectional area of neurons

The cross-sectional area of NM neurons was measured at a higher magnification (40× objective) for estimating the neuronal size. The outline of each neuron at its largest point was traced and the cross-sectional area was determined by ImageJ. Only those neurons with a recognizable nucleolus surrounded by a well-defined nucleus and stained cytoplasma were examined [35], [36]. At least 120 neurons (i.e. >20 neurons in a given section) were measured throughout the rostral to caudal extent of NM on each side in each animal.

### Analysis of CaBP Immunostaining in NM Neurons

Analysis of CaBP immunostaining was performed in a one-in-six series of sections throughout the rostral to caudal extent of NM. Immunofluorescence images were captured with a digital camera (AxioCam MRm, Zeiss) mounted on a fluorescence microscope (Axio Observer Z1, Zeiss) using 20× or 40× objective. The uniform image-acquisition settings (e.g. exposure time) in a given magnification for each antibody was applied throughout the experiments. Because immunostaining intensity is related to antigen concentration [28], [37], the mean gray value was used to indirectly measure the CaBP concentration within NM neurons with the help of ImageJ. It was defined as the sum of the gray values of all the pixels in a neuron divided by the number of pixels. The sum of the gray values of all the pixels in a neuron (the integrated density), which is equivalent to the product of the stained area and the mean gray value, was also calculated for evaluating the amount of CaBPs within NM neurons. Before automated processing, immunostained profiles were extracted by applying a threshold, which was adjusted to obtain the best visual fit between the stained profiles in the original image and those extracted on the screen [26]. Only neuronal soma profiles with a visible nucleus in NM were selected, and the mean gray value, as well as the integrated density in these soma profiles was automatically computed together with the profile number. The same threshold settings were used for each antibody throughout the experiments. An average mean gray value, as well as an average integrated density of all neurons observed on each side of NM was then calculated in each bird. The difference in the average mean gray value, the average integrated density and the soma profile number between the ipsilateral and contralateral sides was expressed as the percentage for the contralateral side in each bird.

### Statistics

All data were expressed as mean ± SEM. The derived ratio data in measurements of immunostaining analysis were evaluated among four groups (control, day 3, 7 and 14 after surgery) using a nonparametric overall analysis (Kruskal-Wallis) followed by post-hoc pairwise comparisons made with Mann-Whitney *U*-tests [35]. For measurements of nucleus volume and cross-sectional area of neurons, paired *t*-test was used to evaluate the difference between the two sides of NM in each group. The mean values in each measurement on the contralateral side were compared among groups using one-way ANOVA.

## Results

### Distribution of CaBPs in NM with Intact Auditory Inputs

**Figure 1:**
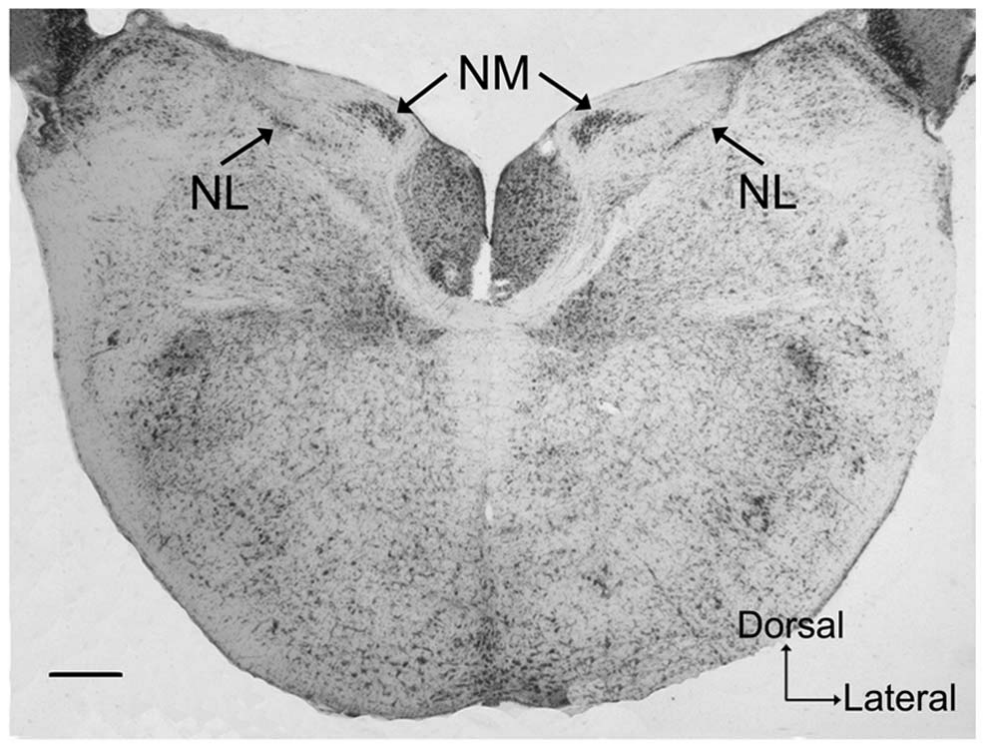
Representative photomicrograph showing a cresyl violet-stained coronal section through NM and NL. The localization of the NM and the NL is indicated by the arrows. Scale bar = 200 µm.

**Figure 2:**
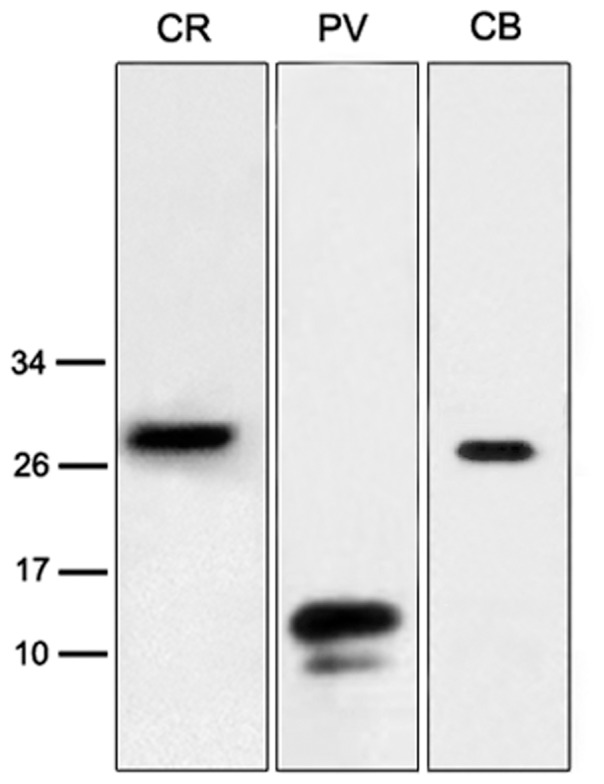
Western blots for CR, PV and CB in zebra finch brain tissue. Positions of molecular weight markers (kDa) are indicated on the left. The major immunoreactive bands were detected at estimated molecular weights of 29 kD, 12 kD and 28 kD for CR, PV and CB, respectively.

The locations of NM and NL in zebra finches were identified using Nissl staining as illustrated in Fig. 1. Western blot analysis of whole brain tissues confirmed specificity of CR, PV and CB antibodies, with an estimated molecular weight of 29 kDa for CR, 12 kDa (the major band) for PV and 28 kDa for CB (Fig. 2, Fig. S1).

**Figure 3:**
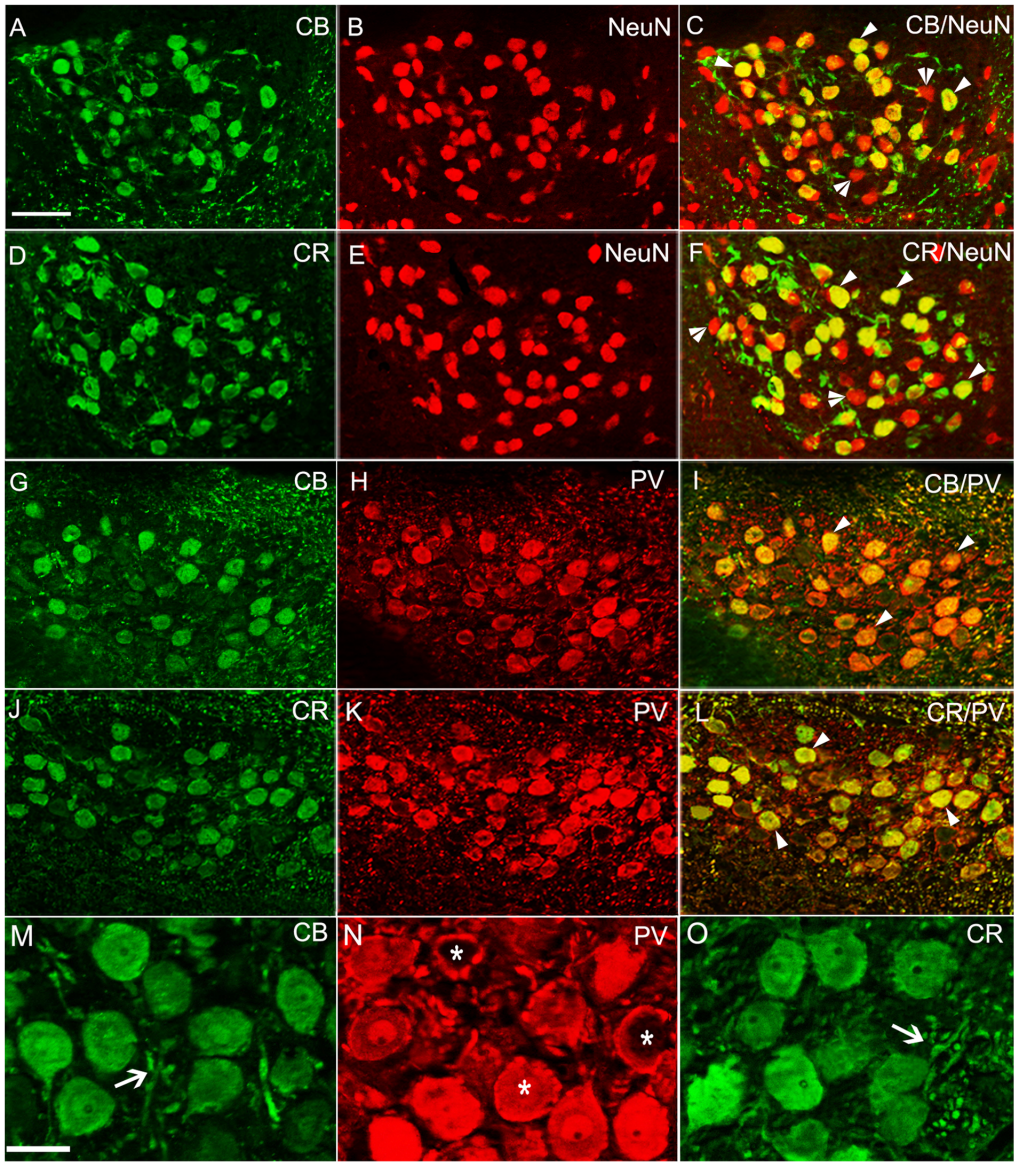
Fluorescent double labeling of CaBPs in NM. **A, G** and **D**, **J** show CB and CR immunoreactivity, respectively. **B**, **E** and **H**, **K** show NeuN and PV immunoreactivity, respectively. **C**, **F**, **I**, **L** show double-labeled immunostaning (Scale bar, 50 µm). **M, N, O** show a higher magnification of CB, PV, CR staining, respectively (Scale bar, 20 µm). The arrows indicate CB or CR positive fibers. The single arrowheads and two arrowheads indicate double labeled neurons and the neurons without CB or CR staining, respectively. The asterisks mark the PV positive puncta that formed distinct perisomatic rings on the immunostained or unstained neurons.

By double immunofluorescence labeling the neuronal marker, NeuN, with CB or CR, we found most neurons in NM expressed these two CaBPs (CB^+^: 77.0±2.4%; CR^+^: 77.9±2.9%, Fig. 3, A–F). However, we also noted presence of a few neurons without any CB or CR staining (Fig. 3, C and F). By calculating the ratio of the total PV positive cells to all NeuN positive cells in a one-in-six series of sections, we could estimate that about 88.6±3.3% neurons were PV immunoreactive. Positively stained CB and CR fibers were also enriched in NM (Fig. 3, M and O). Consistent with the reports in bats and rats [38], [39], some densely stained PV puncta formed distinct perisomatic rings on the immunstained or unlabeled neurons (Fig. 3, N). We also detected the co-localization between CaBPs by double staining PV with CB or CR. A large portion of PV staining cells was CB (86.6±2.9%) or CR positive (88.3±1.9%, Fig. 3, G–L).

**Figure 4:**
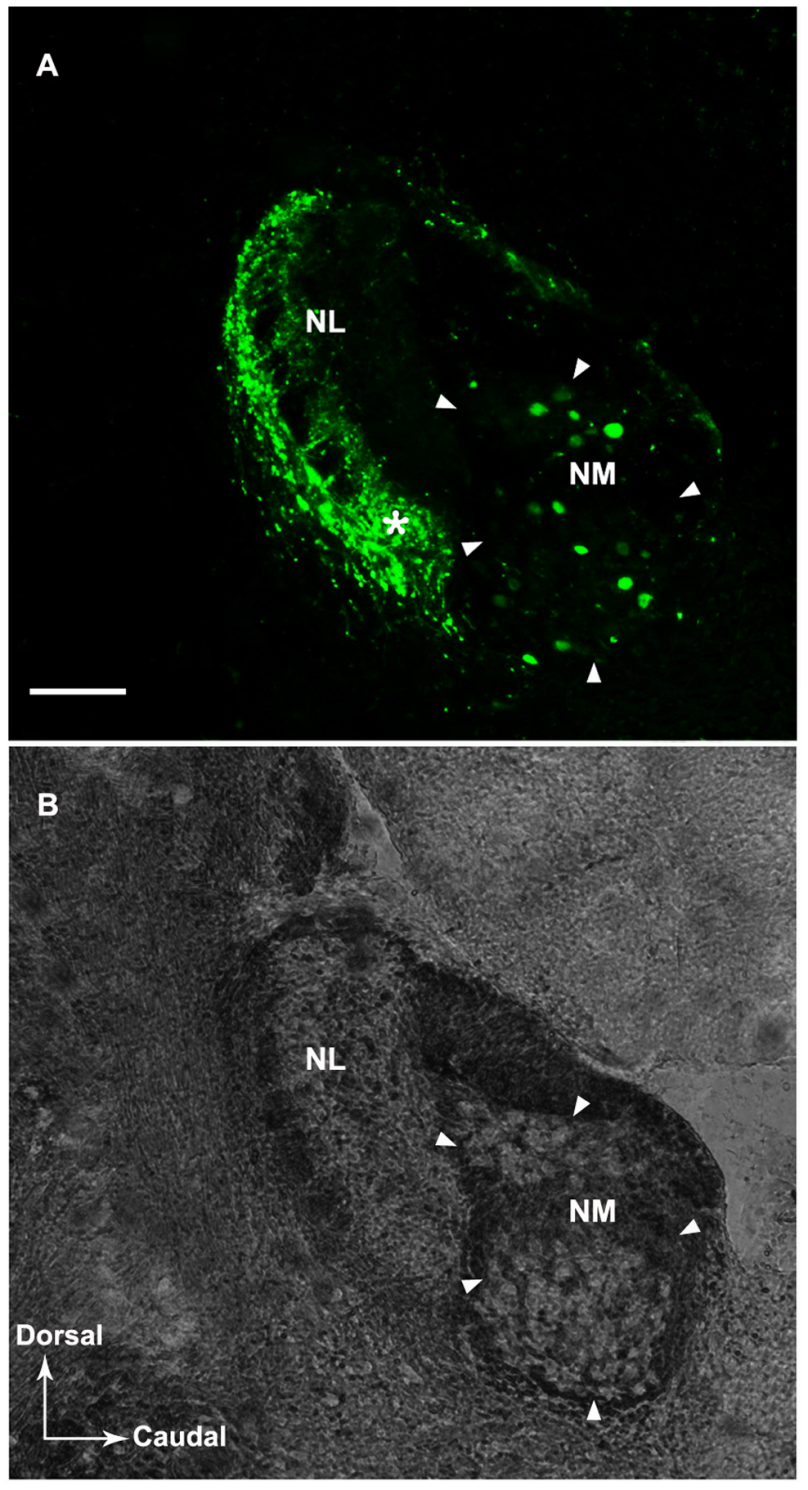
A: An injection of Alexa Fluor 488 dextran in NL at about the middle level along the medial-lateral axis. The tracer distributed throughout the dorsal (rostral) to the ventral (caudal) part of NL at this level. The asterisk marks the approximate injection center. The injection site involved the axons of NM. Retrograde labeling of cell bodies in NM is shown. B: A bright field counterpart of panel A shows the localization of NL and NM. The NM border is indicated by arrowheads. Scale bar = 100 µm.

**Figure 5:**
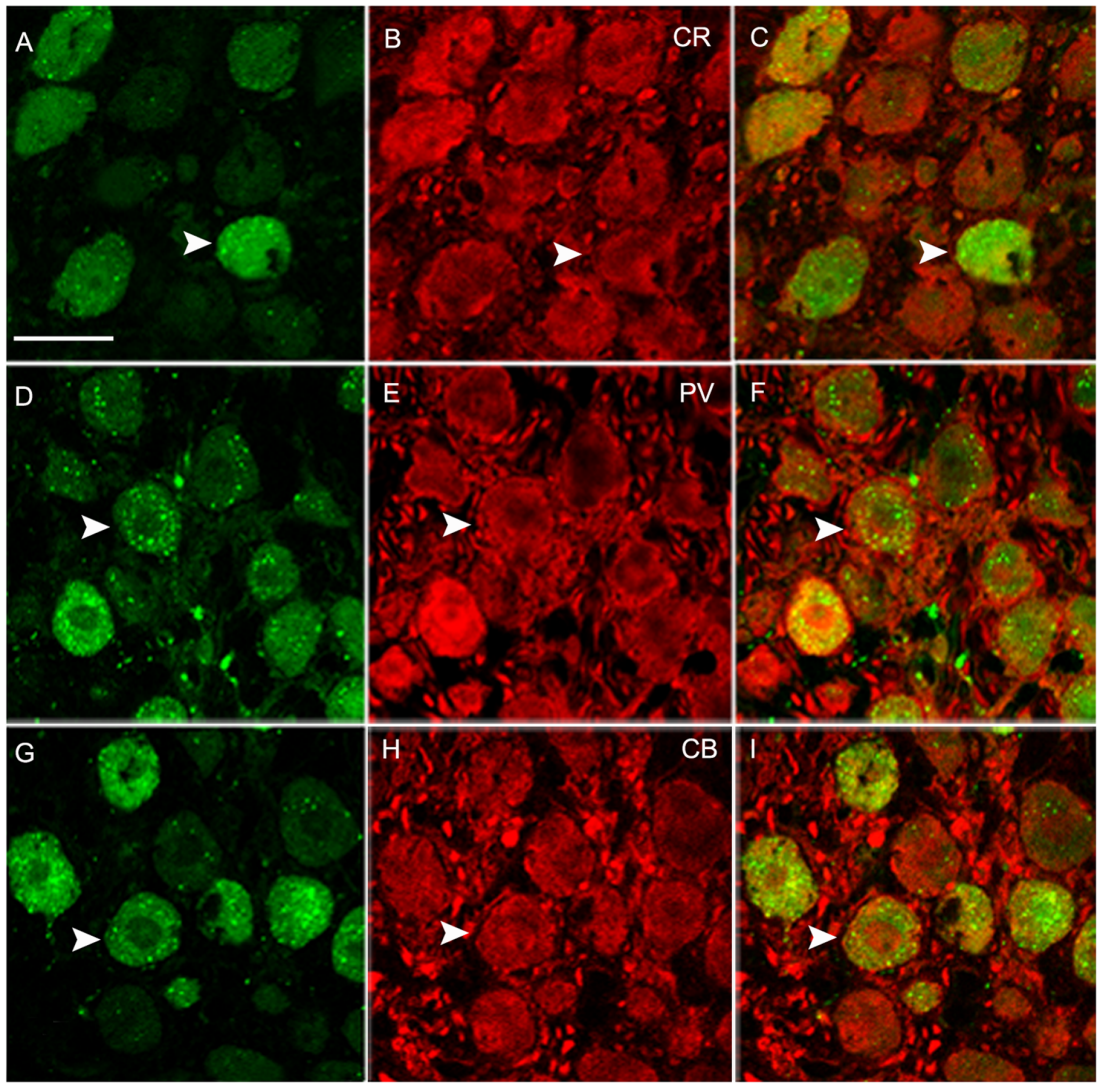
Staining for CaBPs within backfilled NM neurons by a NL injection in single optical sections. **A, D, G** show retrograde labeling. **B, E, H** show CR, PV and CB positive staining, respectively. **C, F, I** show double labeling. The arrowheads indicate the double-labeled cells. Scale bar = 20 µm.

We further confirmed the expression ratios of the three CaBPs in NM by combining immunostaining with retrograde tracing. By injection of Alexa fluor 488 into the NL on one side, many retrogradely filled neurons in the ipsilateral and contralateral NM were clearly observed (Fig. 4, 5). The results of the double labeling of CR, PV or CB with the neuronal tracer showed that most backfilled NM neurons expressed all three CaBPs, similar to the results above. The double labeling ratios were 83.9±0.8%, 88.3±4.0% and 84.3±2.8% for CR, PV and CB, respectively. Control injections outside of NL (i.e. rostral to NL) did not produce any retrograde labeling in NM. Thus, the retrograde labeling of cells in NM is specific due to the injection of tracers into NL, which is consistent with the previous reports in zebra finches [11], [12].

### Histological Alterations in NM following Unilateral Cochlear Ablation

**Figure 6:**
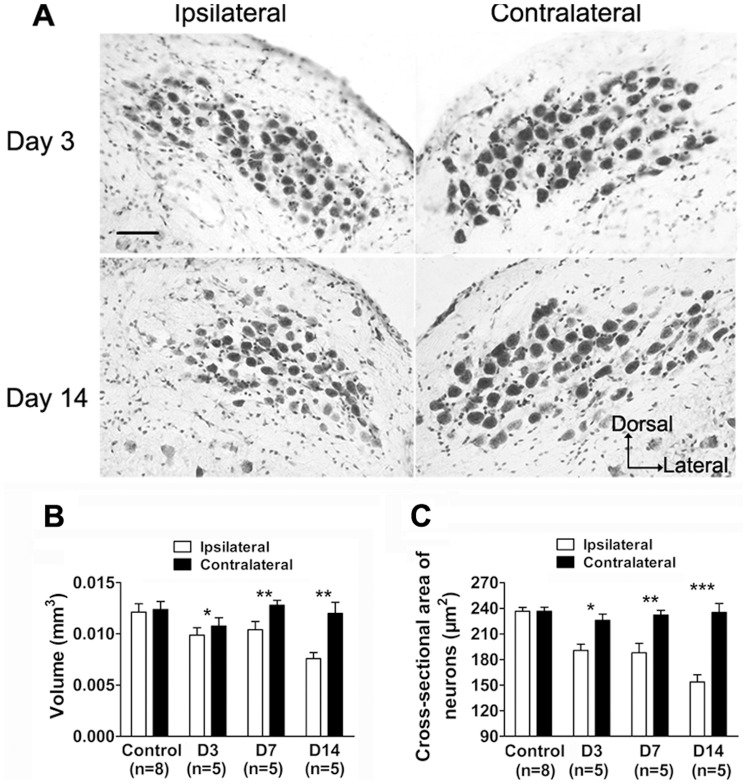
Histological changes in NM following unilateral cochlear removal. **A:** Photomicrographs of cresyl violet-stained coronal sections on the ipsilateral and contralateral side at day 3 or 14 after surgery. Scale bar = 50 µm. **B**, **C:** Bar graphs show the mean values of NM volume and the cross-sectional area of neurons, respectively, on the ipsilateral and contralateral side of different groups. The significant difference between two sides is denoted (*p<0.05, **p<0.01, ***p<0.001). Error bars indicate SEM.

To examine the effects of deafferentation on NM, we performed unilateral cochlear ablation and measured NM volume and cross-sectional area of NM neurons in the context of unilateral cochlear ablation. The volume of the ipsilateral NM reduced significantly at 3 days after surgery (8.0%, p<0.05, paired *t*-test, Fig. 6, B) and further decreased by 36.7% (p<0.01, paired *t*-test, Fig. 6, A and B) at 14 days after surgery. Accompanied with the reduction in volume, cross-sectional area of ipsilateral NM neurons reduced by 15.6% (190.59±7.38 µm^2^, p<0.05, paired *t*-test, Fig. 6, A and C) at 3 days after surgery, and further shrank by 34.6% (153.73±8.36 µm^2^, p<0.001, paired *t*-test, Fig. 6, A and C) at 14 days following surgery. No differences in nucleus volume and cross-sectional area of neurons appeared between the two sides of the control group (p>0.05, paired *t*-test, Fig. 6, B and C). The two measurements on the contralateral side among different groups showed no differences (p>0.05, one-way ANOVA).

### Effects of Unilateral Cochlear Ablation on CaBP Expression in NM Neurons

**Figure 7:**
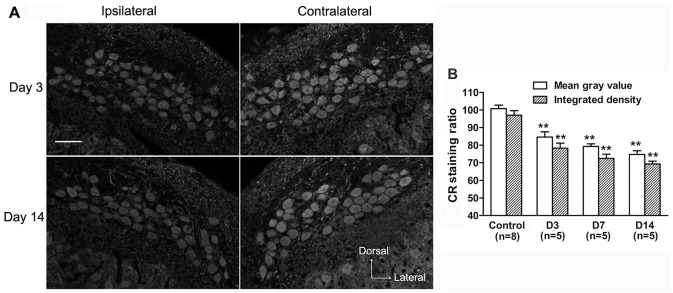
Effects of unilateral cochlear removal on CR expression in NM neurons. **A:** Digital images show CR immunostaining in the ipsilateral and contralateral NM at day 3 and day 14 after surgery. Scale bar = 50 µm. **B:** The bar graph shows the mean ratio of average mean gray value and integrated density of CR staining within neurons of different groups. The significant difference between a surgery and the control group is indicated (**p<0.01). Error bars indicate SEM.

**Figure 8:**
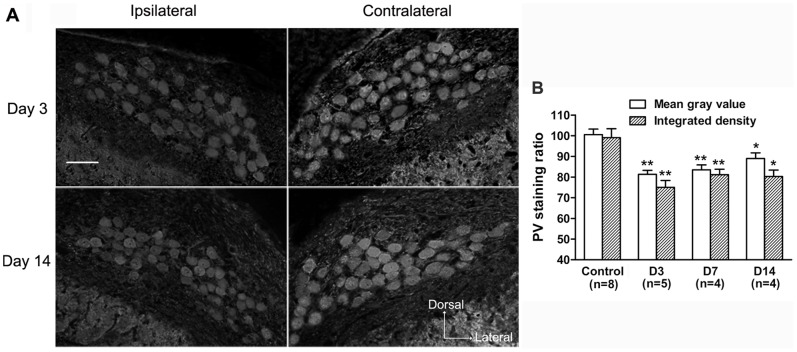
Effects of unilateral cochlear removal on PV expression in NM neurons. **A:** Digital images show PV immunostaining in the ipsilateral and contralateral NM at day 3 and day 14 after surgery. Scale bar = 50 µm. **B:** The bar graph shows the mean ratio of average mean gray value and integrated density of PV staining within neurons of different groups. The significant difference between a surgery and the control group is denoted (*p<0.05, **p<0.01). Error bars indicate SEM.

**Figure 9:**
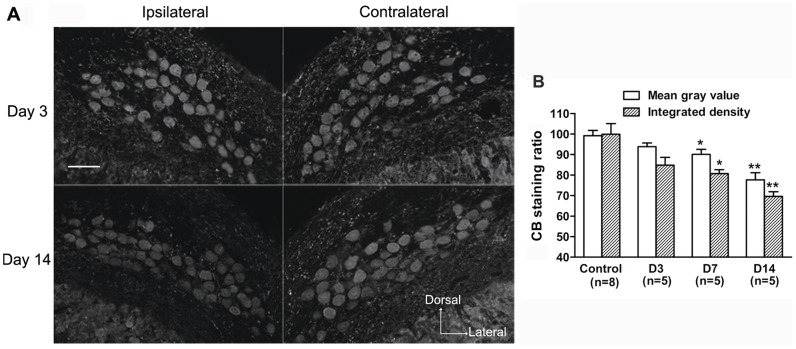
Effects of unilateral cochlear removal on CB expression in NM neurons. **A:** Digital images show CB immunostaining in the ipsilateral and the contralateral NM at day 3 and day 14 after surgery. Scale bar = 50 µm. **B:** The bar graph shows the mean ratio of average mean gray value and integrated density of CB staining within neurons of different groups. The significant difference between a surgery and the control group is indicated (*p<0.05, **p<0.01). Error bars indicate SEM.

Based on the investigations of the distribution of the three CaBPs with intact auditory inputs, we further examined the changes in expression of these CaBPs within NM neurons in response to deafferentation. Following unilateral cochlear ablation, the CR staining within neurons declined rapidly on the ipsilateral side (Fig. 7, A and B). Compared with the control group, the ratio of mean gray value and integrated density significantly decreased to 84.6±3.0% and 78.3±2.9%, respectively (p<0.01, Mann-Whitney *U*-test, Fig. 7, B) at 3 days after surgery, and further reduced to 74.8±2.1% and 69.3±1.6%, respectively (p<0.01, Mann-Whitney *U*-test, Fig. 7, B) at 14 days after surgery. Changes in the PV staining in the ipsilateral NM neurons appeared to fluctuate after cochlear removal (Fig. 8, A and B). Following a rapid decrease at 3 days after surgery (mean gray value: 81.3±1.9%; integrated density: 75.1±3.3%, p<0.01, Mann-Whitney *U*-test, Fig. 8, B), the ratio of mean gray value and integrated density recovered to 89.0±2.8% and 80.3±3.1%, respectively, at 14 days after surgery, which were still lower than the control levels (p<0.05, Mann-Whitney *U*-test, Fig. 8, B). However, the CB staining within neurons in the ipsilateral NM changed relatively slower. Compared with the control group, both ratios first declined at 7 days after surgery (mean gray value: 90.1±2.5%; integrated density: 80.7±2.0%, p<0.05, Mann-Whitney *U*-test, Fig. 9, A and B), and further decreased at 14 days following surgery (mean gray value: 77.7±3.5%; integrated density: 69.6±2.3%, p<0.01, Mann-Whitney *U*-test, Fig. 9, A and B). The number of ipsilateral immunostained soma profiles per slice was significantly decreased at 14 days after surgery (81.6%, 81.3% and 80.1% for CR, PV and CB, respectively; p<0.05, Mann-Whitney *U*-test), compared with control animals. On the contralateral side, there were no obvious changes in the mean gray value and integrated density within NM neurons, and in the number of stained soma profiles for all three CaBPs among different groups (p>0.05, one-way ANOVA).

## Discussion

Our findings provide important details concerning the patterns of CaBP expression and their responses to auditory deprivation in NM in adult zebra finches. We show that PV and CB expression in zebra finch NM are different from that in chickens. In addition, although CR, PV and CB are co-expressed, they show unique temporal plasticity patterns in response to cochlear ablation, indicating they likely play distinct roles within the same neurons in association with auditory processing and transmission in zebra finch NM.

### CaBP Expression Patterns in NM

CaBPs have been applied as excellent markers for specific functional auditory pathways. Previous studies demonstrated that different species exhibit different patterns of CaBP distribution in the auditory brainstem [23], [24], [25], [40], [41]. But to date, there are limited data available for CaBP expression in the brainstem auditory nuclei in songbirds. To our knowledge, only one early study by Braun in 1990 mentioned it briefly and our results confirmed the findings of the high level of PV in zebra finch NM [42]. However, in contrast to the weak CB staining in that report [42], we found most NM neurons densely stained by the antibody against CB under the present conditions. This difference in CB staining may be due to the different efficiency of the antibodies. The CaBP antibodies used in the present study, including that of CB, were demonstrated to be highly sensitive and specific by the western blotting experiment. Other reports also showed high efficiency of this class of CB antibody in several other species, such as mouse, gerbil and zebra finch [43], [44], [45]. CR expression in zebra finch NM was previously unknown.

Extensive data on the patterns of CaBP expression in the cochlear nucleus have currently been reported in non-songbirds and mammals. Our results of high CR levels in zebra finch NM are consistent with those in non-songbirds and most other mammals studied, including barn owl [46], chicken [31], [47], meriones [24], bat [38], rat [48] and guinea pig [25]. However, the patterns of PV and CB expression in NM in zebra finches were quite different from chickens, but similar to some mammals. In chickens, PV and CB staining in NM soma were undetectable [30], [31], [42], [47]. While in most mammals studied, such as guinea pig [25], rat [48] and bat [38], PV labeling was abundant in most neurons and neuropil in the cochlear nucleus, but CB staining was devoid in spherical cells in AVCN, the counterpart of avian NM cells [24], [25], [38], [49], except in chinchilla [50].

We further examined the co-localization patterns of the three CaBPs. Since CR/PV or CB/PV double labeling cells accounted for most of the neurons in NM, we deduced that the majority of NM neurons co-expressed CR, PV and CB. Thus, on the basis of the immunostaining pattern of the three CaBPs, we could not discriminate different neuronal populations in zebra finch NM. This is consistent with the previous knowledge that NM neurons in birds are homogeneous and similar to the spherical bushy cells in the mammalian AVCN [16], [18], [25], [51]. However, there are a few neurons without CR or CB labeling in zebra finch NM. Their neurochemical properties need to be further characterized. Our findings are similar to the recent studies in turtles that CB and PV are co-localized in almost all of neurons in the nucleus cochlearis magnocellularis (CoM), the counterpart of NM [41], [52].

### Histological Changes in Adult Zebra Finch NM Following Auditory Deprivation as Compared with Other Species

Much evidence in both young mammals and chickens shows that histological changes in cochlear nuclei after auditory deprivation, including the reduction in the volume of the nuclei, cell size and cell number, are age-dependent [36], [53], [54], [55], [56], [57]. But compared to mammals, in which the sensitive or critical period for deafferentation closes around the onset of hearing, the window in chickens remains open long after the onset of hearing [58], [59]. Even in adult chickens, neuronal cross-sectional area and cell number are still susceptible to cochlear removal [35], [60]. Consistent with the findings in adult chickens, cochlear ablation induced a reduction of neuron size in NM and the volume of NM in adult zebra finches. Interestingly, Kubke *et al.* found that a chronic hearing loss in the Belgian Waterslager (BWS) canary, a special canary strain which has on average 30% fewer hair cells on the basilar papilla compared to the non-BWS canary, resulted in a significant reduction in cross-sectional area of NM cells and the volume of NM [34]. Previous reports demonstrated the postsynaptic NM neuronal shrinkage to be at least partly, due to the absence of primary auditory nerve axons and decrease of neuronal activity [61], [62]. The decline of ipsilateral NM volume in zebra finches was considered to be partly due to the shrinkage of cells. These results suggest that the mature auditory system in zebra finches continues to maintain a high degree of dependence on auditory stimulation.

### Plasticity Patterns of CaBPs in Response to Deafferentation in Zebra Finch NM as Compared with other Species

In the present study, obvious decreases in the expression of CR, PV and CB within NM neurons in zebra finches occurred in response to the removal of auditory inputs. These alterations are unique in zebra finches, compared with most species studied.

More studies have focused on the changes in CR levels than that in PV and CB in the cochlear nucleus after deafferentation. In chickens, CR expression in NM was independent of auditory inputs [30], [31], in contrast to our findings that CR staining of NM neurons rapidly decreased ipsilaterally in zebra finches. In young adult ferrets, only a small downregulation of CR immunostaining was observed within ipsilateral AVCN neurons [28]. While similar to our results, in adult guinea pigs, the CR immunoreactivity of deafferented AVCN neurons decreased [26]. Regarding the responses in PV levels to deafferentation, only one report by Caicedo *et al.* showed that PV staining within deafferented AVCN neurons in guinea pig increased at first, but was followed by a decrease [26]. On the contrary, we found a down-regulation of PV staining followed by a slight recovery in zebra finches. The considerable differences in CR and PV expression in response to cochlear removal in different species indicate that CR and PV might play different roles among these species or reflect different changes in intracellular events including the calcium levels after deafferentation.

The effects of cochlear removal on CB expression in NM in birds or its counterpart nuclei in other species were largely unknown before, since CB was almost absent in them in most of the species studied [24], [25], [48], except in chinchillas and turtles [41], [50]. It is interesting to notice that there are common hearing characters among the chinchillas, turtles and songbirds, along with CB expression. The chinchilla has exceptionally good low frequency hearing, like humans [50], [63], and turtles also perceive sound in the low frequency range [41]. In songbirds, low frequency but not high frequency auditory feedback is sufficient for song development and maintenance [64]. Such evidence suggests that CB might be associated with the processing of certain specific auditory properties (e.g. low frequency) in these animals. The decline of CB levels within NM neurons in zebra finches after cochlear removal indicates that CB expression is indeed functionally responsive to afferent activity in songbirds. Further investigations will be needed to access the explicit role of CB in NM neurons in songbirds.

### Auditory Plasticity in Songbird NM and Adult Song Plasticity

Much evidence shows that auditory experience continues to be necessary for the maintenance of stereotyped adult songs in zebra finches and Bengalese finches, two most-studied oscine species [4], [6], [65], [66]. For example, bilateral cochlear removal in zebra finches induces subtle but significant changes in syllable structure within a few days to 2 weeks after surgery [67], [68]. In reversibly deafened Bengalese finches, Woolley and Rubel found that as their hair cells regenerated, normal song behavior could be restored; and during this period, adult birds also acquired song material from other birds [5]. So these songbird species exhibit extensive vocal plasticity in adults with response to the altered auditory input. But how does auditory experience shape vocal behaviors? The study in BWS canaries shows that an inherited hearing loss at high frequencies results in significant histological changes in NM and NL [34]. These central auditory alterations may be related to the special loud, low-pitched song behaviors in BWS canaries. In the present study, we also found that conspicuous histological and neurochemical changes occurred as early as at 3 days after hearing loss within NM, appeared in the similar time-course to the onset of vocal degeneration in adult zebra finch as reported [67], [68]. In addition, the changes in CaBP expression in NM after auditory deprivation appeared different from that in chickens, a non-vocal learning species. Therefore, adult vocal plasticity in songbird zebra finches seems to be associated with the plasticity in auditory centers in a species-specific pattern. The research of auditory plasticity in songbirds and other vocal learners may shed light on the mechanisms underlying the learned vocal plasticity.

## Supporting Information

Figure S1 **Effects of the quantity of protein loading in western blot detecting of CB.** An unspecific upper weak band occurred at relatively higher loading quantity (see left lane). This band was diminished, or disappeared when loading quantity was low (see middle and right lane). The protein quantity is indicated below each lane.
(TIF)
