# Supplementary figures and images for: Alteration of CaBP Expression Pattern in the Nucleus Magnocellularis following Unilateral Cochlear Ablation in Adult Zebra Finches
Source: PLoS One. 2013 Nov 14;8(11):e79297. doi: 10.1371/journal.pone.0079297 (PMC3828381; doi:10.1371/journal.pone.0079297)

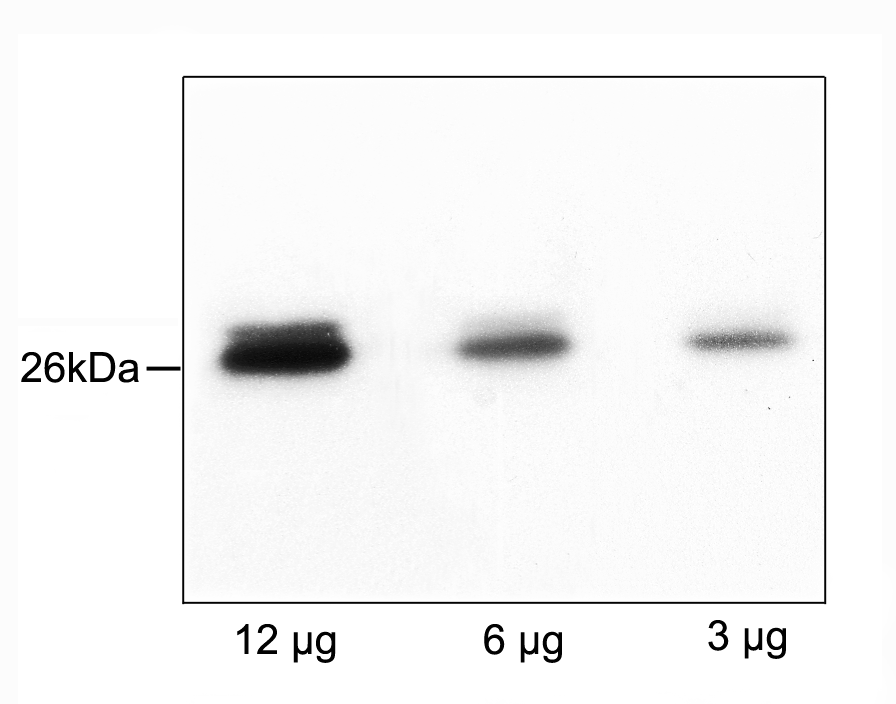

Supplement: Figure S1 — Effects of the quantity of protein loading in western blot detecting of CB. An unspecific upper weak band occurred at relatively higher loading quantity (see left lane). This band was diminished, or disappeared when loading quantity was low (see middle and right lane). The protein quantity is indicated below each lane. (TIF) [file pone.0079297.s001.tif]
